# Light intensity affects the uptake and metabolism of glycine by pakchoi (*Brassica chinensis* L.)

**DOI:** 10.1038/srep21200

**Published:** 2016-02-17

**Authors:** Qingxu Ma, Xiaochuang Cao, Lianghuan Wu, Wenhai Mi, Ying Feng

**Affiliations:** 1 Ministry of Education Key Lab of Environmental Remediation and Ecosystem Health, College of Environmental and Resource Sciences, Zhejiang University, Hangzhou, 310058, China; 2 Zhejiang Provincial Key Laboratory of Subtropic Soil and Plant Nutrition, College of Environmental and Resource Sciences, Zhejiang University, Hangzhou, 310058, China; 3 State Key Laboratory of Rice Biology, China National Rice Research Institute, Hangzhou 310006, China

## Abstract

The uptake of glycine by pakchoi (*Brassica chinensis* L.), when supplied as single N-source or in a mixture of glycine and inorganic N, was studied at different light intensities under sterile conditions. At the optimal intensity (414 μmol m^−2^ s^−1^) for plant growth, glycine, nitrate, and ammonium contributed 29.4%, 39.5%, and 31.1% shoot N, respectively, and light intensity altered the preferential absorption of N sources. The lower ^15^N-nitrate in root but higher in shoot and the higher ^15^N-glycine in root but lower in shoot suggested that most ^15^N-nitrate uptake by root transported to shoot rapidly, with the shoot being important for nitrate assimilation, and the N contribution of glycine was limited by post-uptake metabolism. The amount of glycine that was taken up by the plant was likely limited by root uptake at low light intensities and by the metabolism of ammonium produced by glycine at high light intensities. These results indicate that pakchoi has the ability to uptake a large quantity of glycine, but that uptake is strongly regulated by light intensity, with metabolism in the root inhibiting its N contribution.

The in-depth debate about the forms of nitrogen (N) plants acquire from soils that occurred a century ago is central to our understanding of plant functions in ecosystems and the regulation of plant nutrition. Since the first documentation of preferential absorption and use of organic N by a non-mycorrhizal vascular plant^1^, several studies have shown that plants, including those living in subtropical regions, can take up amino acids and protein directly, thus bypassing the microbial mineralization of the traditional paradigm^2^,^3^,^4^,^5^. Intact amino acids account for more than 50% of plant N in some low temperature ecosystems, such as arctic, alpine tundra, boreal forest, and heathland ecosystems^1^,^6^,^7^,^8^. Moreover, they constitute approximately 0.5–21% of rice, wheat, maize, and tomato N under simulation conditions in the laboratory^5^,^9^,^10^,^11^. Strong evidences have shown that plants have the ability to uptake and utilize organic N, but the quantitative description of organic N contribution under the natural environment is still lacking.

Many biotic and abiotic factors affect plant growth and N uptake, such as nutrition availability, available N forms, mycorrhizae, light intensity, temperature, soil texture, plant species, and unique environmental adaptations that allow absorption of amino acid^4^,^12^,^13^,^14^,^15^. Several studies have shown that plants with large biomass and growth rate tend to take up more inorganic N than that with small biomass, whereas slow-growing plants tend to absorb more organic N than fast-growing plants^15^,^16^. Inorganic and organic N absorption was affected by temperature^17^, and arbuscular mycorrhizae had a considerable effect on the uptake of amino acids and facilitated the uptake of neutral and positively charged amino acids to a greater extent than negatively charged amino acids^18^. Godlewski *et al*. demonstrated that the roots of higher plants can secrete proteases, suggesting that some plant species may have developed a strategy for actively increasing the availability of free amino acids^19^. Furthermore, a ^15^N-labelling study of grasslands in Germany showed that different plant functional groups relied on different N pools to meet their N demands, suggesting that N uptake patterns across functional groups are driven by different fundamental niches^20^, and spatiotemporal variations affects the uptake of organic and inorganic nitrogen^12^.

Light is one of the most important factors mediating plant growth^21^ and is a vital regulator of numerous processes^13^. Light is the energy source for photosynthesis, producing the ATP and NADPH to assemble carbon atoms into organic molecules. Carbon assimilation provides the carbon skeletons needed for nitrogen assimilation, but competes with nitrogen assimilation for ATP and NADPH. Comparisons of the relative uptake of ammonium, nitrate, and amino acids, show that uptake of one form of nitrogen has an effect on the uptake of other forms^17^,^22^. Ammonium hindered the uptake of nitrate, and the external addition of amino acids inhibited the uptake of both ammonium and nitrate^23^. Considering differences between N sources in the energy demands of assimilation and the interactions between N forms, light intensity has a great effect on the uptake of N^24^.

Light intensity varies considerably, regardless of the latitude, day or night, altitude, or ecological niche of plant species. However, it is unclear which forms of N plants acquire under different light intensities, especially organic forms of N. Xingliang studied the effects of light on short-term competition for nitrate, ammonium, and glycine between maize and rhizosphere microorganisms^11^; it was demonstrated that high levels of light intensity significantly increased maize uptake of ^15^NO_3_^−^ and glycine ^14^C, but did not significantly affect the uptake of ^15^NH_4_^+^ or ^15^N-glycine. This suggests that light intensity may alter the competitive relationships between rhizosphere microorganisms and maize roots. However, there is a lack of detailed research exploring the effect of light intensity on the uptake of amino acids and the influence mechanism of it.

Pakchoi (*Brassica chinensis* L.) is one of the most important vegetables in China, planted over a large north-south range^25^. We used pakchoi as our test material, and cultivated it in a sterile environment. The objectives of this study were to (1) determine whether amino acids can play an important role in N nutrition for pakchoi in a sterile environment, (2) research the effect of light intensity on the relative uptake of organic and inorganic N by pakchoi, and (3) reveal the mechanism by which pakchoi alters its uptake of glycine under different light intensities.

## Results

### Pakchoi biomass and long-term N uptake under single or mixed N sources

**Figure 1:**
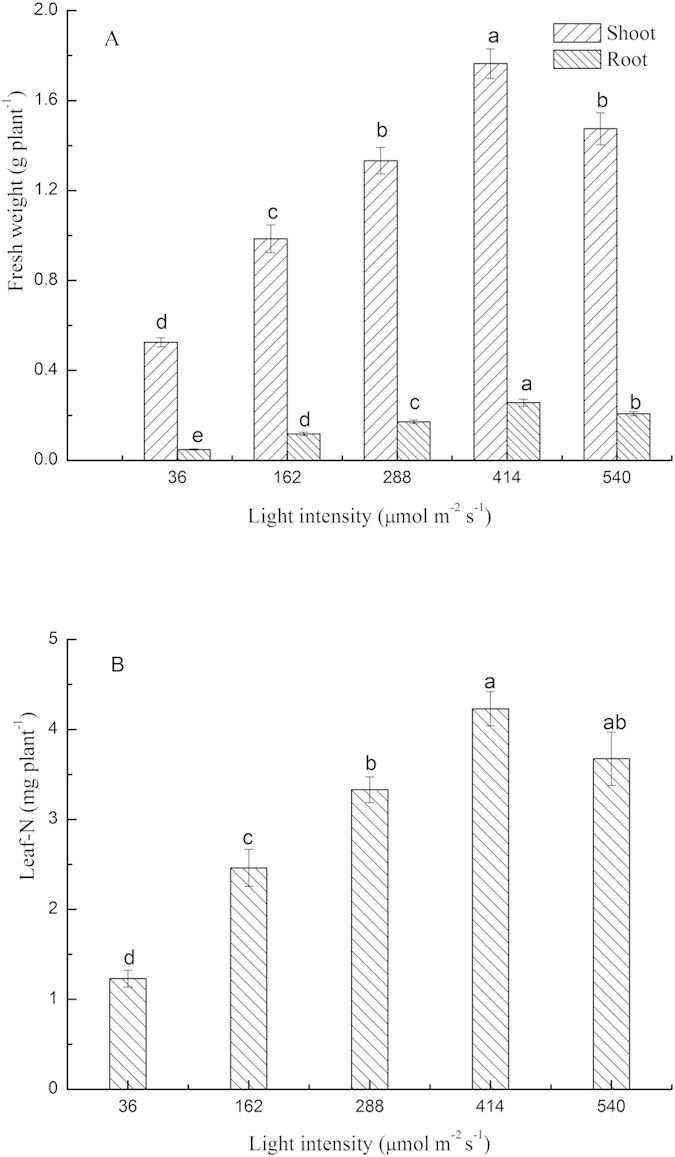
The root and shoot fresh weights (**A**) and leaf-N amounts (**B**) of pakchoi (*Brassica chinensis* L.) under different light intensities. Bars show mean values ± SE, n = 18.

Under the mixed N sources, light intensity had a significant effect on N uptake by pakchoi and plant growth (Fig. 1 p < 0.05). The fresh weight of the shoot and root increased with light intensity up to 414 μmol m^−2^s^−1^. Specifically, the fresh weight of plants grown under 540 μmol m^−2^ s^−1^ light was significantly lower than that of plants grown under 414 μmol m^−2^ s^−1^ light (Fig. 1A). N uptake followed a similar trend to that of pakchoi growth. A curve fitted to light intensity and total N uptake data (mg plant^−1^) yielded the following equation: y = −2E − 05x^2^ + 0.0151x + 0.5961 (R^2^ = 0.95^**^), showing that the optimal light intensity for pakchoi N uptake was approximately 360 μmol m^−2^ s^−1^ (Fig. 1B).

**Figure 2:**
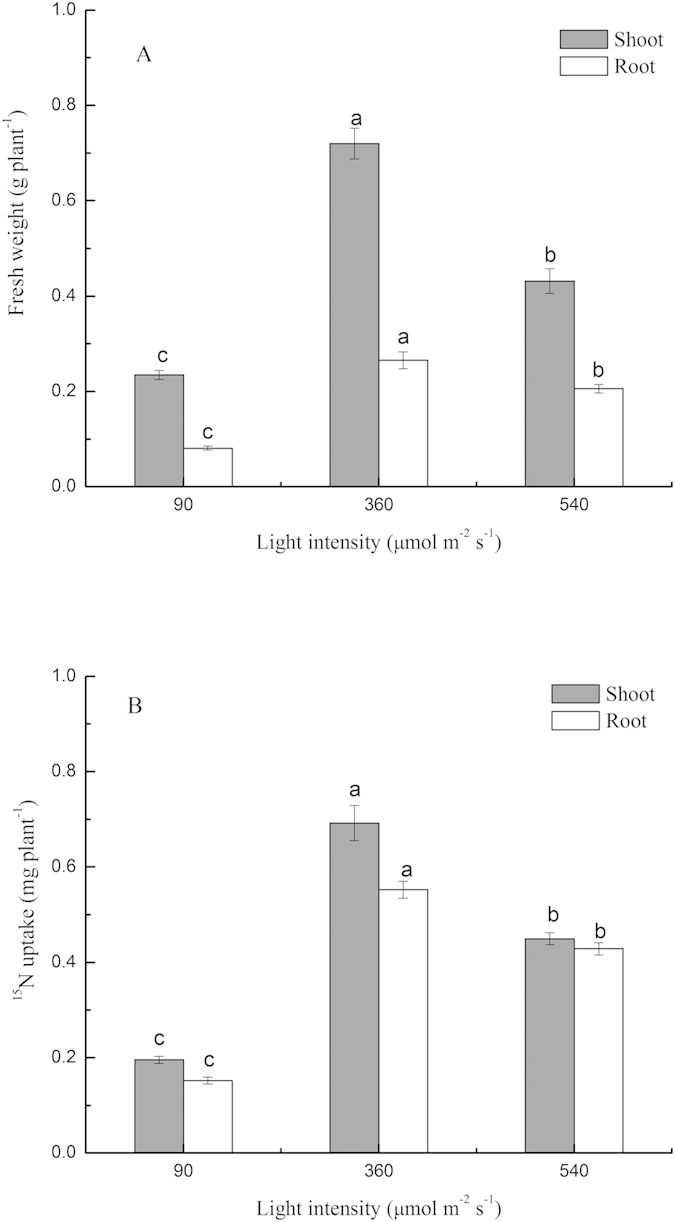
The effect of light intensity on growth (**A**) and glycine-^15^N long-term uptake (**B**) of pakchoi (*Brassica chinensis* L.) in single N sources of glycine. Bars show mean values ± SE, n = 6 for A, and n = 3 for B.

Under the single N source of 3 mM glycine, pakchoi growth and glycine-^15^N uptake were significantly affected by light intensity (Fig. 2 p < 0.01). The biomass and ^15^N uptake of the shoot and root under 360 μmol m^−2^ s^−1^ light were significantly higher than 90 and 540 μmol m^−2^ s^−1^ light, with similar results being obtained for the combined N sources (Fig. 2B).

### Photosynthetic characteristics of pakchoi

Light had a strong effect on the photosynthetic characteristics of pakchoi leaves (Table 1). The photosynthetic rate, conduction to H_2_O, and transpiration rate all increased with light intensity, and peaked at 414 μmol m^−2^ s^−1^; these values were slightly lower in plants grown under 540 μmol m^−2^ s^−1^ light compared to plants grown under 414 μmol m^−2^ s^−1^ light. Intercellular CO_2_ concentration showed an opposite trend, decreasing with increasing light intensity. The lowest intercellular CO_2_ concentration was found in plants grown under 414 μmol m^−2^ s^−1^ light.

### Uptake of glycine, nitrate, and ammonium

**Figure 3:**
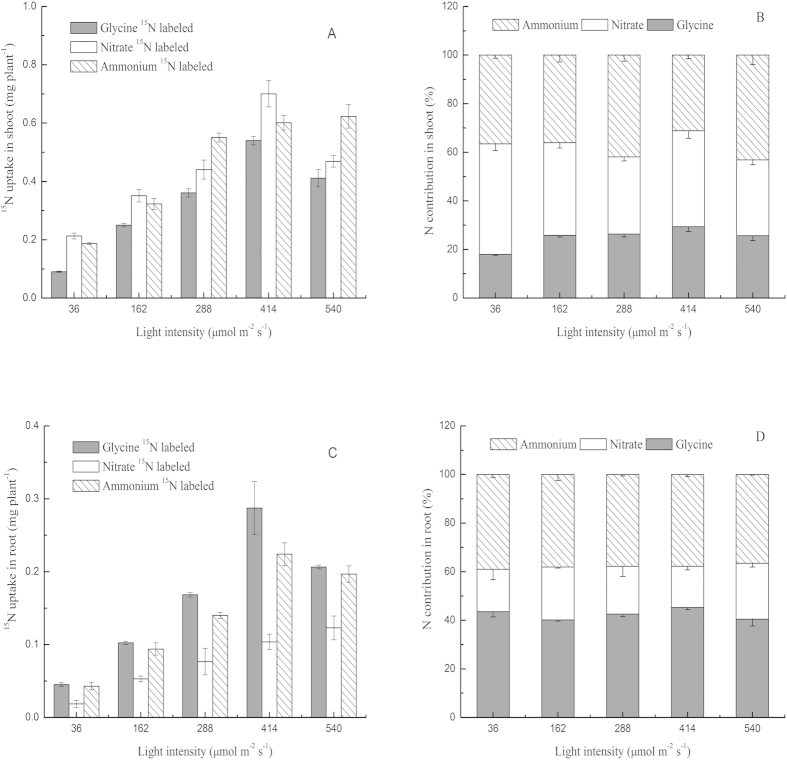
The effect of light intensity on ^15^N uptake of pakchoi (*Brassica chinensis* L.) in multiple N sources. The uptake of glycine, nitrate, and ammonia in shoot (**A**) and root (**C**); and the N contribution of each form of N to total N uptake (%) in shoot (**B**) and root (**D**). Bars show mean values ± SE, n = 3.

Light intensity had a strong effect on the uptake of different N forms (Fig. 3). Shoot ^15^N from glycine and nitrate increased with increasing light intensity (up to 414 μmol m^−2^ s^−1^), while ammonium-^15^N in the shoots under 414 μmol m^−2^ s^−1^ light was similar to those under 288 and 540 μmol m^−2^ s^−1^ light (Fig. 3A). In the shoots, nitrate-^15^N uptake was significantly higher than glycine-^15^N uptake by pakchoi, accounting for 31–45% total N (Fig. 3B). In comparison, nitrate-^15^N was significantly lower than glycine-^15^N in the roots, with the former only accounting for 17–23% and the latter 40–45% total N (Fig. 3D). Furthermore, the N contribution of glycine, nitrate, and ammonium for pakchoi shoots under 36 μmol m^−2^ s^−1^ light were 18%, 45%, and 36%, respectively. In comparison, these values were 29%, 39%, and 31% under 414 μmol m^−2^ s^−1^ light, respectively (Fig. 3B). Glycine was an important source of N for pakchoi, accounting for 18–29% total shoot N uptake, and peaking at 414 μmolm^−2^ s^−1^ light (Fig. 3B).

### Short-term uptake and transformation of glycine

**Figure 4:**
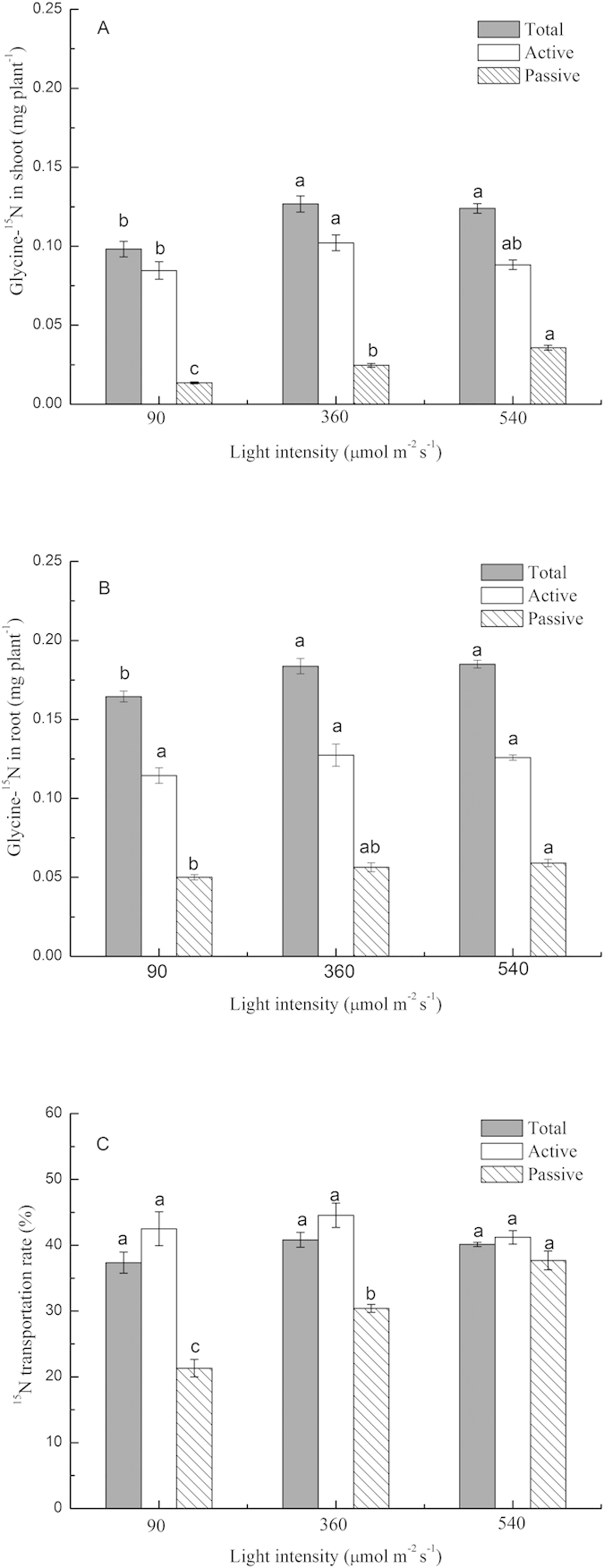
The effect of light intensity on glycine-^15^N short-term uptake by pakchoi (*Brassica chinensis* L.). Glycine-^15^N in shoots (**A**) and roots (**B**), and glycine-^15^N transportation ratio (**C**). Bars show mean values ± SE, n = 3.

Light intensity affected the uptake and root to shoot transportation of glycine-N (Fig. 4). Total uptake and active uptake of glycine-^15^N under optimum light intensity (360 μmol m^−2^ s^−1^, for N uptake) were greater than the uptake of these parameters documented under lower light intensity. In comparison, there was no significant difference in uptake between optimum and over-high light intensity (540 μmol m^−2^ s^−1^). Although active uptake under the over-high light intensity was slightly lower than that documented at the optimum light intensity, the uptake and transportation from root to shoot of passive uptake glycine-^15^N were significantly higher than that under the optimum light intensity (Fig. 4C). The passive uptake of glycine-^15^N increased with light intensity (Fig. 4A,B).

### Activity of glycine metabolic enzymes

GPT enzyme activity in the shoots and GS activity in the shoots and roots varied with light intensity (Table 2). In contrast, the activities of GPT in roots, and GOT in shoots and roots showed no significant difference among the different light-intensity treatments. GS activity in the shoots and roots under optimum light intensity was higher compared to over-high light intensity.

### Amino acids contents of pakchoi

**Figure 5:**
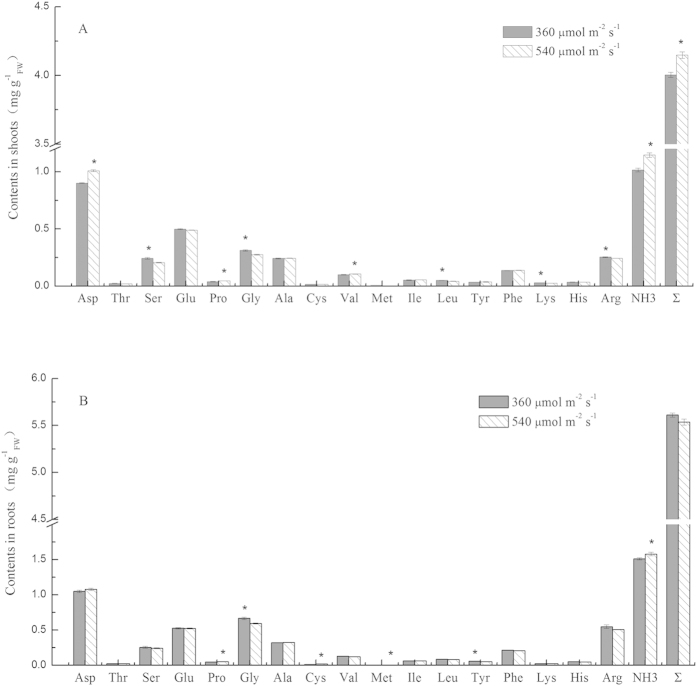
The effect of light intensity on the contents of amino acids and ammonia in pakchoi shoots (**A**) and roots (**B**) with a 12 h uptake of 3 mM glycine. Bars show mean values ± SE, n = 6.

The amino acids and NH_3_ in the shoots and roots differed under optimum light (360 μmol m^−2^ s^−1^) and over-high light (540 μmol m^−2^ s^−1^) over a 12 h period (Fig. 5). In the shoots, the contents of aspartic acid, proline, and valine under over-high light were significantly higher than optimum light. In contrast, serine, glycine, leucine, lysine, and arginine contents were lower under over-high light compared to optimum light (Fig. 5A). Under the over-high light, the roots contained more proline, cysteine, and methionine, with lower glycine and tyrosine than under optimum light (Fig. 5B). Seedlings that grew under over-high light contained more NH_3_ in the shoots and roots, with the sum of the amino acids and NH_3_ in the shoots being significantly higher than that under optimum light conditions (Fig. 5A,B).

## Discussion

### Effect of light intensity on pakchoi growth

Light energy is used to produce NADPH and ATP in the light reactions of photosynthesis and is important for plant growth^26^. The biomass of pakchoi roots and shoots increased with increasing light intensity up to 414 μmol m^−2^ s^−1^. Under low light intensity, carbohydrate biosynthesis and carbon fixation are limited by ATP availability^27^, whereas excessive light intensity generates oxygen radicals and causes photoinhibition^28^. Both of these conditions seriously limit primary productivity. The highest light intensity used in our experiments was unlikely to cause severe stress, as the intensity of sunlight may reach around 2000 μmol m^−2^ s^−1^ in the middle of the day^29^. However, when exposed to a constant intensity of light for 12 h each day, the optimum light intensity for pakchoi seedling growth was approximately 400 μmol m^−2^ s^−1^, with 540 μmol m^−2^ s^−1^ light having a negative effect on growth over 25 days of culture. The light saturation point differs among plant species, as shown by Carrigan, who reported that 16-h daily exposure to 1000 μmol m^−2^ s^−1^ light has a passive impact on tomato growth, altering leaf morphology and stomatal behaviour, as well as reducing plant height and photosynthesis rates^13^.

### Effect of light intensity on N uptake

Light intensity had a strong effect on the uptake of N and different N forms. Under controlled growth conditions with constant light intensity throughout the day, 360 μmol m^−2^ s^−1^ was the optimum light intensity for N uptake. Ammonium is not the preferred N source for most plant species; however, the N contribution of ammonium was higher in the shoots than those of nitrate and glycine under 288 and 540 μmol m^−2^ s^−1^ light. This result indicates that light intensity changed preferred N uptake by pakchoi. Most plants tend to take up nitrate, even though ammonium may require less processing than nitrate. Nitrate taken up by roots must be converted to ammonium by nitrate reductase, and additional metabolic processes are required for the synthesis of amino acids^30^. However, nitrate taken up by roots was mostly transported to shoots, whereas ammonium was assimilated to amino acids in the roots^31^. The difference in the metabolism process had a great effect on N uptake. Optimum light intensity provided enough energy, in addition to improving the passive uptake and transportation of N, which increased the uptake of nitrate. The uptake of different nitrogen was regulated by many factors, with the single factor analysis showing that light intensity is important.

Pakchoi absorbs nitrate to a greater degree than ammonium or organic N^32^. In our study, the quantity of ^15^N-nitrate in pakchoi shoots exceeded that of other N forms under optimum light intensity; however, the ^15^N-nitrate in the pakchoi roots was much lower than other N forms. In addition, the N contribution of nitrate in the shoots had a significant negative correlation with its N contribution in the roots. Research clearly showed that nitrate taken up by the roots is mostly transported to the shoots, whereas ammonium is assimilated to amino acids in the roots^31^. Under different light intensities, we documented 1) lower ^15^N-nitrate in the roots, but higher uptake in shoots, 2) higher ^15^N-glycine content in the roots, but lower content in the shoots, and 3) greater change in the contribution of each N form in the shoots compared to the roots. These results indicate that, first, most ^15^N-nitrate uptake by the root was rapidly transported to the shoot, with the pakchoi shoot having a powerful assimilative capacity. Second, N contribution of glycine was not limited by uptake, but was limited by assimilation ability or the transportation of amino acids and its production in the roots. Third, light intensity affected the N contribution of different N forms by regulating the metabolism of leaves, rather than root uptake.

### N contribution of glycine

Plants have the ability to take up and metabolize a large number of amino acids. In the present study, glycine accounted for 18%–29% total N uptake over 25 days of cultivation. As the amino acids transporters that have been identified in most crops^33^,^34^, the uptake rates of organic N were similar to those of inorganic N^35^; thus, organic N should not be overlooked as an important source of N for crops. However, the test was completed in a sterilized environment, bypassing the competition of microorganism, which is considered to be more competitive for organic N. Furthermore, low concentrations of amino acids in soil solutions limit the N-contribution of amino acids . However, soil amino acids have fast turnover rates (i.e., a few hours); hence, the flux of amino acids into plants maybe large, despite low concentrations in soil solution^17^. Whether amino acids have an unrecognized or negligible role in plant nutrition could not be determined, because accurate method to determine the quantitative contribution of organic nitrogen is still lacking^36^. However, we showed that pakchoi possesses the ability to uptake and metabolism a large quantity of glycine, which warrants further investigation.

There is evidence of direct organic N uptake by plants in various plant community types^34^. Many of these ecosystems occur in low-temperature zones, such as the arctic^37^, alpine tundra^22^, boreal forest^38^, and heath lands^1^,^39^, where there is a lack of inorganic N and relatively slow mineralization of organic N. Mean light intensity differs markedly with latitude, and is higher in temperate, subtropical, and tropical zones than boreal areas. In the present study, glycine contributed a substantial proportion of total N (up to 29%) in pakchoi, which was higher under high light intensity than under low light intensity. However, in the natural environment, high temperature occurs in parallel with high light intensity in most cases, which promote strong organic N mineralization. Roots were regarded as stronger competitors for inorganic N than microorganisms, whereas microorganisms were strong competitors for organic N^11^. So, increasing light intensity caused the plant uptake of amino acids to increase, but a decline in microorganism activity. The effect of light intensity and the interaction effect of light and temperature on the uptake of organic N in natural environments require more detailed research.

### Potential mechanisms for the effect of light intensity on glycine uptake

The N contribution of glycine was highest under the optimum light intensity in both mixed nitrogen sources and a single source of glycine. This result may be due to energy support from photosynthesis, increased glycine uptake and transport, or increased assimilation of glycine N.

In the present study, light intensity had a strong effect on the photosynthetic characteristics of plants. For plants cultivated at 540 μmol m^−2^ s^−1^ light, the photosynthesis rate was lower than that of plants cultivated at 414 μmol m^−2^ s^−1^ light; however, the intracellular CO_2_ concentration was relatively low in both plant groups. We speculated that the low internal CO_2_ concentration in plants grown at 414 μmol m^−2^ s^−1^ light was caused by the consumption of CO_2_ during photosynthesis. In contrast, for plants grown under 540 μmol m^−2^ s^−1^ light, the observed low internal CO_2_ concentration was caused by the closure of leaf stomata. Thus, photosynthesis was limited by high light intensity. This phenomenon may have inhibited the active uptake of amino acids (Fig. 4A), as this process requires energy.

The majority of amino acids absorbed by plants are taken up through a series of co-transporters that are driven by H^+^-ATPase^40^. This result is consistent with our observation that glycine absorption in CCCP un-treated plants was 3.3- to 4.1-fold greater than that of CCCP-treated plants, because CCCP inhibits active uptake of glycine. Although active uptake represented approximately 70% of total absorbed glycine, passive uptake also contributed to glycine uptake, especially at optimum and high light intensities. The active and passive uptake of glycine in plants exposed to optimum light intensity was significantly higher than those in plants exposed to low light intensity. This result indicates that uptake was likely the rate-limiting step for glycine utilization in low intensity light. Over a 10-d culture period, glycine uptake was significantly higher under optimum light intensity than under over-high light intensity. However, in the short-term uptake test, the active uptake of glycine was similar in plants exposed to optimum and high light intensity, whereas the passive uptake of glycine was greater in plants exposed to over-high light intensity than in those exposed to optimum light intensity, indicating that uptake was not the limiting step under the over-high light intensity.

N contribution is regulated by uptake, transportation, and assimilation. The long-term uptake test shown that glycine uptake in over-high light was significantly lower than optimum light (Fig. 2B), but the short-term uptake of glycine over-high light was not lower than optimum light (Fig. 4A,B), which may indicating that glycine metabolism is critical for its contribution over long periods. The glutamine synthetase (GS)/glutamate synthase (GOGAT) cycle is considered the major pathway for N assimilation and regulation of nitrogen metabolism in higher plants^41^. Reduced glutamine synthetase activity is important for controlling photosynthetic responses to high intensity light in leaves^42^. This phenomenon is consistent with the relatively low GS activity that was observed in plants under over-high light intensity in the present study. These findings are further supported by the fact that amino acid uptake significantly decreases after exposure to the GS inhibitor methionine sulphoximine (MSX)^43^. Thus, glycine metabolism may be the rate-limiting step for glycine absorption in plants under high intensity light.

Although there is broad consensus that plants absorb organic N, little is known about the metabolism and distribution of amino acids following uptake, and the regulation of these processes^36^,^43^. A previous study showed that glycine taken up by the roots was primarily metabolized via transaminase reactions, with partial ^15^N labelled glycine being detected in xylem sap^44^. In contrast, another study showed that glycine-N was probably metabolized by deamination in roots. Specifically, while U-^13^C, ^15^N-glycine was not detected in xylem sap^36^. The circulation of GS-GOGAT is also important way for organic nitrogen metabolism. For instance, amino acids taken up by ryegrass are transformed to other amino acids by transamination, with the generated ammonium being assimilated to free amino acids by GS-GOGAT^45^. Persson suggested that alanine taken up by Scots pine (*Pinus sylvestris* L.) root is transformed to other amino acids by alanine aminotransferase or Ala-2-oxo acid transaminase^43^. Unfortunately, we were unable to clarify this phenomenon here. However, based on the concentration of amino acids and ammonium in the shoots and roots, we detected major differences in serine and ammonium concentrations in the shoots and roots of pakchoi under optimum or over-high light conditions. These results may provide week evidence for the coexistence of the two pathways.

Several studies have shown that plants take up amino acids at relatively high rates. Thus, it is useful to investigate the bottlenecks for plant growth performance on amino acids^43^, and to study the bottlenecks under various environments. Amino acid uptake is regulated by the root concentrations of ammonium, with uptake declining after exposure to GS inhibitor MSX^43^. Our results were consistent with this previous study, where we showed that higher concentrations of ammonium were correlated with lower GS activity at the highest light intensity. Higher ammonium and lower GS activity may indicate that ammonium metabolism is a limiting step at over-high light intensity. Thornton detected ^15^N in amino acids derived from ^15^N-Glycine by gas chromatography-mass spectrometry, showing that uptake is the limiting step at low temperatures, while the metabolism of glycine to serine is the limiting step at high temperatures^17^. The sum of amino acids and ammonium at over-high light intensity was similar or higher than that under optimum light. This result showed that uptake is not the limiting step at over-high light intensity, supporting the short-term uptake results. At over-high light intensity, serine and glycine concentrations in the shoots and roots were lower than those under the optimum light intensity. This result indicates that light had little effect on the transamination of glycine, and the accumulated ammonium may come from deamination of glycine. Proline is a signaling molecule that regulates mitochondrial functions and cell proliferation or death, and is critical in the process of plant stress adaptation and recovery, signal transduction, and removal of free radicals, which are essential processes for plant recovery from stress^46^. High proline content in seedlings at over-high light intensity may with adaptation to light stress, showing that 540 μmol m^−2^ s^−1^ light was too great for pakchoi growth.

### Significance of studying the influence of light intensity on N uptake

In our 25-d absorption test, the contribution of different N forms to total N differed greatly among tested light intensities. The contribution of glycine to total N ranged from 18% to 29%, indicating that light intensity had a significant effect on the relative uptake of different N forms, in addition to its influence on plant growth and total N uptake. However, previous studies placed limited focus on the lighting conditions of test environments during short-term N absorption tests and controlled-condition tests, and many fail to provide clear descriptions about lighting conditions^5^,^9^,^18^,^47^. Furthermore, the optimum light intensity for N uptake varies among species, with the uptake of different N forms changing greatly. Lighting conditions that differ from the optimal light intensity may result in the overestimation or underestimation of the contributions of glycine-N to total N in short-term tests. Furthermore, differences in the lighting conditions used for short-term tests make it difficult to compare the contributions of glycine-N to total N across studies.

It is worthwhile to study and predict the environmental factors that influence N uptake and plant growth, to enhance ecosystem research and management. Since the climate is changing greatly with the effect of global warming, greater focus should be placed on the effect of environmental change, allowing greater flexibility in managing ecosystems, especially the agriculture ecosystem. Although the effects of pH^48^,^49^, light intensity^11^, and temperature^50^ on the uptake of ammonium, nitrate, and/or amino acids have been studied independently, the interaction of these factors has received limited research. Thus, general models to predict the compounding effects of environmental factors are needed in the face of the future climate change.

## Methods

Pakchoi (Zhebai 6) was cultivated in a sterile environment. Pakchoi seeds were soaked for 12 h in purified water, and sterilized as described by Wu *et al*.^51^. The seeds were then sown in sterilized culture dishes containing two layers of moist cotton gauze at the bottom and this was defined as day 1 of each experiment. The culture dishes were placed in a sterilized culture room, with a photoperiod of 12 h and light intensity of 380 μmol m^−2^ s^−1^ provided by fluorescent lamp, a day/night temperature of 25/20 °C and humidity of 60%/40%. Three days after germination, seedlings grew to approximately 1 cm length. Seedlings were transplanted to 50 mL centrifuge tubes (filled with 0.5% cooling-off agar) with small holes in the caps. After 2 days, the seedlings had completely grown out of the holes and the holes were sealed with silicone rubber (Nanda 704). One day later, the seedlings were transferred together with the centrifuge tube cap to a new centrifuge tube (covered with silver paper to avoid any effects of light on root growth), which was filled with nutrient solution. The nutrient solution contained 4 mM CaCl_2_, 2 mM K_2_SO_4_, 2 mM KH_2_PO_4_, 1.4 mM MgSO_4_·7H_2_O, 0.1 μM NaMoO_4_·2H_2_0, 0.4 μM CuSO_4_·5H_2_O, 1 μM ZnSO_4_·7H_2_O, 8 μM H_3_BO_3_, 10 μM MnCl_2_, 5 μM Na_2_EDTA, and 18.3 μM FeSO_4_·7H_2_O, and the pH was adjusted to 6.2. N mixtures prepared for each experiment were added to the nutrient solution before use. The nutrient solution and all materials, except seeds and N solution, were sterilized by steam under high pressure (121 °C for 30 min). The N solutions were passed through a 0.22 μm membrane filter (Millipore, PES Membrane, Ireland) prior to combining with the nutrient solution. The nutrient solution was changed every 3 days during experiments in a clean bench.

### Experiment 1: Effect of light intensity on pakchoi growth, photosynthesis, and relative uptake of nitrate, ammonium, and glycine

Three mixtures of NO_3_^−^, NH_4_^+^, and glycine (1:1:1) were prepared. In each mixture, a different N source was labelled with ^15^N (50.22% Na ^15^NO_3_^−^, 50.17% (^15^NH_4_)_2_SO_4_ or 50.16% ^15^N-glycine). Each N mixture was tested separately. N mixtures were added to the nutrient solution for a total N concentration of 3 mM. Light intensity was controlled by relative distance from the fluorescent lamp, and plants received 36, 162, 288, 414, and 540 μmol m^−2^ s^−1^ light. There were 15 treatments in total (5 light intensity treatments ×3 N mixtures) and 6 pakchoi seedlings were subjected to each treatment. In addition, we created two “blank” seedlings for one treatment by providing it with unlabelled N mixture at the same concentration as for treated plants. After 22 days, 9 pakchoi seedlings were randomly selected from each light intensity treatment. Then the photosynthetic characteristics were measured on fully expanded leaves using a portable open-flow gas exchange system Li-6400 (LI-COR Biosciences, Lincoln, NE, USA). The concentration of CO_2_ was 385 μmol L^−1^, and the light intensity was 1000 μmol m^−2^ s^−1^. On day 25, the pakchoi seedlings were destructively sampled.

### Experiment 2: Effect of light intensity on the long-term uptake of glycine

In experiment 1, we showed that the optimum light intensity for glycine uptake was approximately 360 μmol m^−2^ s^−1^ under a combined nitrogen source; however, this result raised the question of what intensity is ideal when a single N resource (glycine) is available. Pakchoi seedlings were pre-cultivated for 25 days in a sterile environment (as in experiment 1), under the light intensity of 360 μmol m^−2^ s^−1^, with the exception of the N mixtures. For this experiment, N was provided as 3 mM NO_3_^−^, 0.5 mM NH_4_^+^, and 0.5 mM glycine because NO_3_^−^-N accelerates root growth to meet the sample quantity requirements for testing. After pre-cultivation, 24 pakchoi seedlings were cultivated for 10 days under the light intensities of 90, 360, and 540 μmol m^−2^ s^−1^, and N was provided as 3 mM 50.16% ^15^N-glycine, providing an equimolar N concentration with experiment 1. The nutrient solution was changed every 3 days. Based on the results of experiment 1, 360 μmol m^−2^ s^−1^ was the optimal light intensity for glycine uptake by pakchoi, whereas 90 and 540 μmol m^−2^ s^−1^ were too low and too high for pakchoi growth, respectively.

### Experiment 3: Effect of light intensity on the short-term uptake of glycine

Experiment 2 showed that light intensity has a strong effect on glycine uptake, raising the question of whether this phenomenon was caused by uptake. Pakchoi seedlings were pre-cultivated for 25 days in a sterile environment as in experiment 2. After pre-cultivation, 36 similar pakchoi seedlings (6 for each treatment) were selected, and the roots and centrifuge tubes were washed several times with purified water. The seedlings were “hungry” cultivated in sterilized nutrient solution without N for 1 night (approximately10 h), after which a short-term absorption test was performed. Eighteen pakchoi seedlings were cultured with 3 mM-labelled glycine (98.10% ^15^N) under 90, 360, and 540 μmol m^−2^ s^−1^ light for 4 h.

The effects of light intensity on the active and passive absorption of glycine were also examined by using carbonyl cyanide 3-chlorophenylhydrazone (CCCP)^52^ at the same time. Eighteen “hungry” cultivated pakchoi seedlings were then pre-treated with 50 μM CCCP for 1h, and cultured with 98.10% ^15^N glycine under 90, 360, and 540 μmol m^-2^ s^−1^ light for 4 h. The ^15^N in CCCP-treated plants was the result of passive uptake.

Roots and shoots in experiment 1, 2, and 3 were harvested separately, and pairs of seedlings from each treatment group were combined to form single samples. To remove ^15^N on root surfaces, roots were washed by ultrasonification in sterile water, followed by 50 mM CaCl_2_, and several washes with purified water. The roots and aboveground parts were freeze-dried (Labconco Freezen System, USA) and ground to a fine powder with a ball mill (Retsch MM301, German). The N content was determined by the Micro-Kjeldahl method (Yihong, NKD6200), titrated with 0.05 mM sulphuric acid. The titrated solution was condensed by rotary evaporator (EYELA, SB-1100) at 55 °C until N concentration was greater than 0.5 mg mL^−1^. The ^15^N enrichment of the condensed solution was determined using a Tracer MAT-271 (Finnigan MAT, USA).

### Experiment 4: Effect of light intensity on the activity of glycine metabolic enzymes

Experiment 2 showed that long-term glycine uptake in 90 and 540 μmol m^−2^ s^−1^ was significantly lower than 360 μmol m^−2^ s^−1^ light; however, in experiment 3, the short-term uptake of glycine in 540 μmol m^−2^ s^−1^ light was not lower than that in 360 μmol m^−2^ s^−1^ light, raising the question of whether the metabolism of glycine inhibits the N contribution of glycine. Pakchoi seedlings were pre-cultured for 25 days as described in experiment 2. Eight similar size seedlings at each level of light intensity were selected, and the roots were washed several times with purified water. The seedlings were “hungry” cultivated for 1 night, then cultivated with 3 mM glycine for 4 days, and the nutrient solution was changed every 2 days. Glycine was supplied at a concentration of 3 mM to provide an equimolar concentration of N with experiment 1, while excluding the influence of other N forms on the activity of enzymes. The activities of glutamine synthetase (GS)^53^, glutamic-pyruvic transaminase (GPT), and glutamic oxalacetic transaminase (GOT)^54^ in the roots and leaves were measured. Over an extended period, biomass may differ significantly among groups, which may affect the enzyme concentrations in plants because of the “magnification effect” or the “dilution effect.” Therefore, we subjected the seedlings to different light intensities for just 4 days. In this way, the biomass of seedlings did not vary significantly among treatment groups (data not shown), allowing us to conclude that any differences in enzyme activity were due to the effects of light intensity rather than to biomass.

### Experiment 5: Effect of light intensity on the metabolism of amino acids

Experiment 4 showed that glutamine synthetase in 540 μmol m^−2^ s^−1^ light was significantly lower than that in 360 μmol m^−2^ s^−1^ light; thus, the limiting step of amino acid metabolism was investigated here. Pakchoi seedlings were pre-cultured for 25 days, as described in experiment 2. Twenty-four similar size seedlings at each level of light intensity were selected, and the roots were washed several times with purified water. The seedlings were “hungry” cultivated for 14 h. Then, the seedlings were cultivated with 3 mM glycine under 360 and 540 μmol m^−2^ s^−1^ light for 12 h. Subsequently, the roots and shoots of pakchoi were harvested separately, and 4 seedlings from each treatment group were combined to form single samples. To remove glycine from the root surfaces, roots were washed by ultrasonification in sterile water, followed by 50 mM CaCl_2_, and several washes with purified water. Further, 1 g aliquots of fresh shoots or roots were ground with 4 ml 5% sulphosalicylic acid, and left to stand for 1 h. The solution was centrifuged at 14000 g for 10 min. The supernatant was retained and passed through a 0.2 μm membrane filter. Amino acid content was detected by an automatic amino-acid analyser (L-8900, Hitachi, Japan).

### Calculations and statistics

The uptake of different N sources was determined by the ^15^N concentration in treated seedlings relative to the ^15^N concentration in “blank” seedlings not provided with labelled N. The amount of NO_3_^−^, glycine, and NH_4_^+^ taken up from the labelled N was calculated using equation 1,^55^


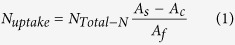


where *N*_*uptake*_is the amount of a given N source taken up into the roots or shoots of pakchoi seedlings; *N*_*Total-N*_ is the total N content of the roots or aboveground parts; *A*_*s*_ is the ^15^N atom% in the roots or aboveground parts; *A*_*c*_ is the ^15^N atom% in the “blank” that was supplied with unlabelled N mixture in the shoot of experiment 1 and the shoot or root of experiment 2; *A*_*f*_ is the ^15^N atom% of the labelled-N source {glycine (50.16%), NO_3_^−^ (50.22%), or NH_4_^+^ (50.17%) for experiment 1 and glycine (98.10%) for experiment 2}.


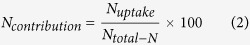


where *N*_*contribution*_ is the proportion of total N taken up as glycine, NO_3_^−^ or NH_4_^+^ by whole pakchoi seedlings; *N*_*uptake*_ is the amount of a given N source taken up into the roots or shoots of pakchoi seedlings, as calculated from equation (1); and *N*_*total-N*_ is the total N total mass of N contained in pakchoi seedlings.


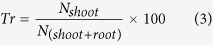


where Tr is the transportation rate; *N*_*shoot*_ is the amount of glycine-N in the shoot; and *N*_*(shoot + root)*_ is the total glycine-N in the root and shoot, as calculated by equation (1).

### Statistical analysis

All statistical analyses were performed using SAS 8.2. Data are presented as the mean ± standard error (SE). One-way analysis of variance (ANOVA) was used to evaluate differences between treatments and differences in mean values were tested using Duncan’s multiple range method (p < 0.05). Figures were created using Origin 8.1.

## Additional Information

**How to cite this article**: Ma, Q. *et al*. Light intensity affects the uptake and metabolism of glycine by pakchoi (*Brassica chinensis* L.). *Sci. Rep.* **6**, 21200; doi: 10.1038/srep21200 (2016).

## Figures and Tables

**Table 1:** The effect of light intensity on the photosynthetic characteristics of pakchoi (*Brassica chinensis* L.).

Light intensity (μmol m^−2^ s^−1^)	Photosynthetic rate (μmol CO_2_ m^−2^ s^−1^)	Transpiration rate (mmol H_2_O m^−2^ s^−1^)	Conductance to H_2_O (mol H_2_O m^−2^ s^−1^)	Intercellular CO_2_ concentration (μmol CO_2_ mol^−1^)
36	7.12 ± 0.36 d	8.06 ± 0.55 c	0.23 ± 0.02 c	527 ± 17.3 a
162	10.44 ± 0.58 c	9.55 ± 0.64 bc	0.31 ± 0.02 b	396 ± 6.7 b
288	10.81 ± 0.72 c	9.70 ± 0.62 bc	0.32 ± 0.02 b	389 ± 4.6 b
414	17.75 ± 1.00 a	11.52 ± 0.58 a	0.42 ± 0.02 a	314 ± 17.5 c
540	12.73 ± 0.33 b	11.20 ± 0.48 ab	0.40 ± 0.03a	314 ± 19.9 c

Values represent the mean ± SE (n = 9). Different letters in each column indicate significant differences between treatments at the p < 0.05 level.

**Table 2:** The effect of light intensity on the activity of glycine metabolic enzymes in *Brassica chinensis* L.

Light intensity (μmol m^−2^ s^−1^)	GPT in shoot (μmol·g^−1^·30 min)	GPT in root (μmol·g^−1^·30 min)	GOT in shoot (μmol·g^−1^·30 min)	GOT in root (μmol·g^−1^·30 min)	GS in shoot (A·mg^−1^ protein·h^−1^)	GS in root (A·mg^−1^ protein·h^−1^)
90	6.1 ± 0.4b	25.8 ± 1.0a	16.9 ± 0.4a	25.6 ± 0.4a	4.4 ± 0.2b	63.7 ± 10.6a
360	8.7 ± 0.7a	22.5 ± 2.6a	15.4 ± 0.4a	25.5 ± 0.6a	5.6 ± 0.2a	40.8 ± 5.7b
520	8.2 ± 0.4a	22.2 ± 1.5a	16.2 ± 0.6a	25.3 ± 0.3a	4.6 ± 0.1b	24.6 ± 1.7c

Values represent the mean ± SE (n = 4). Different letters in each column indicate significant differences between treatments at the p < 0.05 level. Abbreviations: GPT, glutamic-pyruvic transaminase enzyme; GOT, glutamic oxalacetic transaminase; GS, glutamine synthetase.

## References

[b1] Chapin F. S., Moilanen L. & Kielland K. Preferential use of organic nitrogen for growth by a non-mycorrhizal arctic sedge. Nature 361, 150–153 (1993).

[b2] Näsholm T., Huss-Danell K. & Högberg P. Uptake of glycine by field grown wheat. New Phytol 150, 59–63 (2001).

[b3] Miller A. E., Bowman W. D. & Suding K. N. Plant uptake of inorganic and organic nitrogen: neighbor identity matters. Ecology 88, 1832–1840 (2007). 17645029 10.1890/06-0946.1

[b4] Lonhienne T. G. A. . Effects of externally supplied protein on root morphology and biomass allocation in Arabidopsis. Sci rep-UK 4 (2014). 10.1038/srep05055 PMC4031471 24852366

[b5] Ge T. . Amino acids as a nitrogen source for tomato seedlings: The use of dual-labeled (^13^C, ^15^N) glycine to test for direct uptake by tomato seedlings. Environ Exp Bot 66, 357–361 (2009).

[b6] Lipson D. & Näsholm T. The unexpected versatility of plants: organic nitrogen use and availability in terrestrial ecosystems. Oecologia 128, 305–316 (2001). 24549899 10.1007/s004420100693

[b7] Näsholm T., Ekblad A. & Et A. Boreal forest plants take up organic nitrogen. Nature 392, 914–916 (1998).

[b8] Warren C. R. Uptake of inorganic and amino acid nitrogen from soil by Eucalyptus regnans and Eucalyptus pauciflora seedlings. Tree Physiol 29, 401–409 (2009). 19203963 10.1093/treephys/tpn037

[b9] Cao X. . Rice uptake of soil adsorbed amino acids under sterilized environment. Soil Biol Biochem 62, 13–21 (2013).

[b10] Kaštovská E. & Šantrůčková H. Comparison of uptake of different N forms by soil microorganisms and two wet-grassland plants: A pot study. Soil Biol Biochem 43, 1285–1291 (2011).

[b11] Xu X., Stange C. F., Richter A., Wanek W. & Kuzyakov Y. Light affects competition for inorganic and organic nitrogen between maize and rhizosphere microorganisms. Plant Soil 304, 59–72 (2008).

[b12] Gao J., Mo Y., Xu X., Zhang X. & Yu F. Spatiotemporal variations affect uptake of inorganic and organic nitrogen by dominant plant species in an alpine wetland. Plant Soil 381, 271–278 (2014).

[b13] O Carrigan, A. . Effects of light irradiance on stomatal regulation and growth of tomato. Environ Exp Bot 98, 65–73 (2014).

[b14] Shi G. . Interactive influence of light intensity and soil fertility on root-associated arbuscular mycorrhizal fungi. Plant Soil 378, 173–188 (2014).

[b15] Harrison K. A., Bol R. & Bardgett R. D. Do plant species with different growth strategies vary in their ability to compete with soil microbes for chemical forms of nitrogen? Soil Biol Biochem 40, 228–237 (2008).

[b16] Weigelt A., Bol R. & Bardgett R. D. Preferential uptake of soil nitrogen forms by grassland plant species. Oecologia 142, 627–635 (2005). 15549402 10.1007/s00442-004-1765-2

[b17] Thornton B. & Robinson D. Uptake and assimilation of nitrogen from solutions containing multiple N sources. Plant Cell environ 28, 813–821 (2005).

[b18] Whiteside M. D., Garcia M. O. & Treseder K. K. Amino acid uptake in arbuscular mycorrhizal plants. Plos One 7, e47643 (2012). 23094070 10.1371/journal.pone.0047643 PMC3475604

[b19] Godlewski M. & Adamczyk B. The ability of plants to secrete proteases by roots. Plant physiol Bioch 45, 657–664 (2007). 10.1016/j.plaphy.2007.06.001 17761428

[b20] Kahmen A., Renker C., Unsicker S. B. & Buchmann N. Niche complementarity for nitrogen: an explanation for the biodiversity and ecosystem functioning relationship? Ecology 87, 1244–1255 (2006). 16761603 10.1890/0012-9658(2006)87[1244:ncfnae]2.0.co;2

[b21] Rodríguez-García E. & Bravo F. Plasticity in Pinus pinaster populations of diverse origins: Comparative seedling responses to light and Nitrogen availability. Forest Ecol Manag 307, 196–205 (2013).

[b22] Miller A. E. & Bowman W. D. Alpine plants show species-level differences in the uptake of organic and inorganic nitrogen. Plant Soil 250, 283–292 (2003).

[b23] Thornton B. Inhibition of nitrate influx by glutamine in Lolium perenne depends upon the contribution of the HATS to the total influx. J Exp Bot 55, 761–769 (2004). 14754920 10.1093/jxb/erh066

[b24] El-Naggar A., de Neergaard A., El-Araby A. & Gh-Jensen H., H. Simultaneous Uptake of Multiple Amino Acids by Wheat. J Plant Nutr 32, 725–740 (2009).

[b25] Chen W., Luo J. & Shen Q. Effect of NH_4_^+^-N/NO_3_^−^-N ratios on growth and some physiological parameters of Chinese cabbage cultivars. Pedosphere 15, 310–318 (2005).

[b26] Strengbom J., Näsholm T. & Ericson L. Light, not nitrogen, limits growth of the grass Deschampsia flexuosa in boreal forests. Can j Bot 82, 430–435 (2004).

[b27] Solymosi K. & Schoefs B. Etioplast and etio-chloroplast formation under natural conditions: the dark side of chlorophyll biosynthesis in angiosperms. Photosynth Res 105, 143–166 (2010). 20582474 10.1007/s11120-010-9568-2

[b28] Massimo B. . Low-night temperature increased the photoinhibition of photosynthesis in grapevine (Vitis vinifera L. cv. Riesling) leaves. Environ Exp Bot 57, 25–31 (2006).

[b29] Zhong-Hua C. . Systems dynamic modeling of the stomatal guard cell predicts emergent behaviors in transport, signaling, and control. Plant Physiol 159, 1235–1251 (2012). 22635112 10.1104/pp.112.197350 PMC3404696

[b30] G. R. F. A comparative study of ammonium toxicity at different constant pH of the nutrient solution. Plant Soil 103, 239–243 (1987).

[b31] NM, C. & ADM & G. Molecular and physiological aspects of nitrate uptake in plants. Trends Plant Sci 3, 389–395 (1998).

[b32] Wang H., Wu L., Zhu Y. & Tao Q. Growth, nitrate accumulation, and macronutrient concentration of pakchoi as affected by external nitrate-N: amino acid-N ratio. J Plant Nutr 31, 1789–1799 (2008).

[b33] Jones D. L., Healey J. R., Willett V. B., Farrar J. F. & Hodge A. Dissolved organic nitrogen uptake by plants-an important N uptake pathway? Soil Biol Biochem 37, 413–423 (2005).

[b34] Näsholm T., Kielland K. & Ganeteg U. Uptake of organic nitrogen by plants. New Phytol 182, 31–48 (2009). 19210725 10.1111/j.1469-8137.2008.02751.x

[b35] Wallenda T. & Read D. J. Kinetics of amino acid uptake by ectomycorrhizal roots. Plant Cell Environ 22, 179–187 (1999).

[b36] Warren C. R. Post-uptake metabolism affects quantification of amino acid uptake. New Phytol 193, 522–531 (2012). 22007943 10.1111/j.1469-8137.2011.03933.x

[b37] Nordin A., Schmidt I. K. & Shaver G. R. Nitrogen uptake by arctic soil microbes and plants in relation to soil nitrogen supply. Ecology 85, 955 (2004).

[b38] Bennett J. N. & Prescott C. E. Organic and inorganic nitrogen nutrition of western red cedar, western hemlock and salal in mineral N-limited cedar–hemlock forests. Oecologia 141, 468–476 (2004). 15322895 10.1007/s00442-004-1622-3

[b39] Thornton B. & Robinson D. Uptake and assimilation of nitrogen from solutions containing multiple N sources. Plant Cell Environ 28, 813–821 (2005).

[b40] Bush D. R. Proton-Coupled Sugar And Amino Acid Transporters In Plants. Annu Rev Plant Biol 44, 513–542 (2003).

[b41] Zhao W. . Expression, purification, and characterization of recombinant mangrove glutamine synthetase. Mol Biol Rep 41, 7575–7583 (2014). 25086623 10.1007/s11033-014-3649-9

[b42] Brestic M. . Reduced glutamine synthetase activity plays a role in control of photosynthetic responses to high light in barley leaves. Plant Physiol Bioch 81, 74–83 (2014). 10.1016/j.plaphy.2014.01.002 24491798

[b43] Persson J. Uptake, metabolism and distribution of organic and inorganic nitrogen sources by Pinus sylvestris. J Exp Bot 57, 2651–2659 (2006). 16820399 10.1093/jxb/erl028

[b44] Schmidt S. & Stewart G. R. Glycine metabolism by plant roots and its occurrence in Australian plant communities. Aust J Plant Physiol 26, 253–264 (1999).

[b45] Thornton B., Osborne S. M., Paterson E. & Cash P. A proteomic and targeted metabolomic approach to investigate change in Lolium perenne roots when challenged with glycine. J Exp Bot 58, 1581–1590 (2007). 17431027 10.1093/jxb/erl294

[b46] Szabados L. & Savouré A. Proline: a multifunctional amino acid. Trends Plant Sci 15, 89–97 (2010). 20036181 10.1016/j.tplants.2009.11.009

[b47] Scott E. E. & Rothstein D. E. Amino acid uptake by temperate tree species characteristic of low- and high-fertility habitats. Oecologia 167, 547–557 (2011). 21553264 10.1007/s00442-011-2009-x

[b48] Hawkins B. J. & Robbins S. pH affects ammonium, nitrate and proton fluxes in the apical region of conifer and soybean roots. Physiol Plantarum 138, 238–247 (2010). 10.1111/j.1399-3054.2009.01317.x 19947965

[b49] Brix H. Root-zone acidity and nitrogen source affects Typha latifolia L. growth and uptake kinetics of ammonium and nitrate. J Exp Bot 53, 2441–2450 (2002). 12432036 10.1093/jxb/erf106

[b50] Warren C. R. Why does temperature affect relative uptake rates of nitrate, ammonium and glycine: A test with Eucalyptus pauciflora. Soil Biol Biochem 41, 778–784 (2009).

[b51] Wu L. H., Mo L. Y., Fan Z. L., Tao Q. N. & Zhang F. S. Absorption of glycine by three agricultural species under sterile sand culture conditions. Pedosphere 15, 286–292 (2005).

[b52] Persson J. R. & Sholm N., T. Regulation of amino acid uptake in conifers by exogenous and endogenous nitrogen. Planta 215, 639–644 (2002). 12172847 10.1007/s00425-002-0786-5

[b53] Horchani F., Hajri R. & Aschi-Smiti S. Effect of ammonium or nitrate nutrition on photosynthesis, growth, and nitrogen assimilation in tomato plants. J Plant Nutr Soil Sc 173, 610–617 (2010).

[b54] Wu L. H., J S. H. & T Q. N. The application of colormetric method on the determination of transaminase activity. Chin J Soil Sci, 41–43 (1998).

[b55] Sauheitl L., Glaser B. & Weigelt A. Uptake of intact amino acids by plants depends on soil amino acid concentrations. Environ Exp Bot 66, 145–152 (2009).

